# Association between paternal pre-pregnancy body mass index with preterm birth and low birth weight

**DOI:** 10.3389/fped.2022.955544

**Published:** 2022-09-29

**Authors:** Mengting Sun, Senmao Zhang, Letao Chen, Yihuan Li, Jingyi Diao, Jinqi Li, Jianhui Wei, Xinli Song, Yiping Liu, Jing Shu, Tingting Wang, Ping Zhu, Jiabi Qin

**Affiliations:** ^1^Department of Epidemiology and Health Statistics, Xiangya School of Public Health, Central South University, Changsha, Hunan, China; ^2^National Health Committee (NHC) Key Laboratory of Birth Defect for Research and Prevention, Hunan Provincial Maternal and Child Health Care Hospital, Changsha, Hunan, China; ^3^Guangdong Cardiovascular Institute, Guangdong Provincial People’s Hospital, Guangdong Academy of Medical Sciences, Guangzhou, Guangdong, China; ^4^Hunan Provincial Key Laboratory of Clinical Epidemiology, Changsha, China

**Keywords:** pre-pregnancy, body mass index, preterm birth, low birth weight, risk factor

## Abstract

**Background:**

With the current global epidemic of obesity, especially among men, there is a need to understand its impact on adverse pregnancy outcomes. This study aimed to assess whether paternal pre-pregnancy body mass index (BMI) was associated with preterm birth and low birth weight in offspring.

**Methods:**

Multinomial logistic regression model was used to analyze associations between paternal BMI and preterm birth and low birth weight in different subgroups, the final model was adjusted for confounding factors of mothers and fathers. Further subgroup analysis was conducted to explore the stability of the risk associations.

**Results:**

A total of 34,104 participants were included in this study, including 1,442 (4.2%) underweight, 13,930 (40.9%) overweight and 5,008 (14.7%) obese according to paternal BMI. The total incidence of preterm birth was 11.85% (4041/34104), and the incidence of low birth weight was 8.86% (3020/34104). In the total study population, compared with normal weight men, paternal pre-pregnancy overweight or obese was associated with a significantly increased risk of preterm birth [aOR; 95% CI respectively (1.34; 1.25–1.45 vs. 1.26; 1.14–1.40)] and low birth weight [aOR; 95% CI respectively (1.60; 1.46–1.74 vs. 1.40; 1.25–1.58)] in offspring. The results of subgroup analysis showed that the direction of the risk association was consistent, indicating good stability.

**Conclusion:**

Paternal pre-pregnancy overweight and obesity were associated with an increased risk of preterm birth and low birth weight in their offspring.

## Introduction

In recent years, overweight and obesity have increased rapidly worldwide. Almost a third of the world’s population is now classified as overweight or obese (1). It is estimated that by 2030, the global incidence of overweight and obesity will reach 1.3 billion and 573 million people respectively, 43 and 21% of whom will live in Asia (2). The situation in China is also not optimistic. From 1980 to 2015, the prevalence of obesity in China increased nearly nine times (from 0.6 to 5.3%). It is worth noting that both the prevalence of obesity and the combined prevalence of overweight and obesity of men in China increased more rapidly than in women, and their weight continues to increase, especially among men (3). Paternal obesity has been reported to negatively affect sex hormones and sperm quality, such as increased estrogen concentration, sperm DNA fragmentation, mitochondrial membrane potential, etc. (4–6). The decrease of sperm quality will directly affect the signal transmission between sperm and ovum (7), and then affect the health of offspring by interfering with embryo development. Therefore, it is necessary to pay attention to the impact of paternal overweight and obesity on the health of their offspring.

Preterm birth and low birth weight are significant global health problems (8). It was reported that the estimated global preterm birth rate was 10.6% (95% CI: 9.0–12.0) in 2014. Among them, the preterm birth rate was estimated at 12% in China, ranking second in the world (9). According to the World Health Organization (WHO), low birth weight accounted for 20% of all live births worldwide (10). In addition, preterm birth and low birth weight also pose long-term health threats, such as long-term neurological and developmental disorders, motor development disorders, cognitive decline, cerebral palsy, epilepsy, etc. (11–14). These have caused a heavy burden of disease to society and families. Thus, it can be seen that looking for the risk factors of preterm birth and low birth weight, especially the controllable and intervenable environmental factors, to reduce the incidence of diseases has become the focus of attention.

Currently, most studies only focus on the impact of maternal body mass index (BMI) on pregnancy outcomes. Studies have found that overweight and obesity in women may be associated with an increased risk of pregnancy complications and outcomes, such as preterm birth, stillbirth, gestational diabetes, gestational hypertension and birth defects (15–18). However, studies on paternal BMI for pregnancy outcomes are relatively sparse and controversial. A multicenter prospective study found that both paternal obesity and central adiposity were associated with a 60% increase in risk of fathering a small for gestational age (SGA) infant (19). An observational study found a link between paternal pre-pregnancy BMI and their offspring’s birth weight, but not as much as mothers’ (20). There were also studies that have reported that paternal BMI was not associated with preterm birth and low birth weight (21–23).

The uncertainty of research evidence has brought obstacles to disease (preterm birth and low birth weight) prevention. It is well known that paternal pre-pregnancy overweight or obesity is a potentially modifiable and preventable factor for adverse neonatal outcomes. Therefore, we intend to explore the association between the paternal pre-pregnancy BMI and the preterm birth and low birth weight of the offspring through a prospective epidemiological investigation, so as to provide a scientific basis for reducing the incidence of diseases.

## Materials and methods

### Study design and population

The study was a prospective cohort study. The participants were couples who received their first antenatal care in the reproductive center, obstetrics department, infertility clinic, other departments of Hunan Maternal and Child Health Hospital from 13 March 2013 to 31 December 2019, and planned to continue the antenatal care and delivery in the same hospital. This study was approved by the Ethics Committee of Xiangya School of Public Health, Central South University, and registered in the Chinese Clinical Trial Registry (registration number: ChiCTR1800016635). All participants provided written informed consent prior to participating in our study. This study complied with the ethical principles of the Helsinki Declaration of the World Medical Association.

**FIGURE 1:**
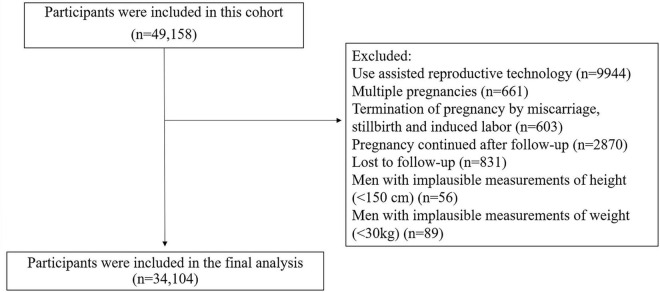
Flow chart showing the process of participant’s recruitment.

In the study period, 49,158 eligible pregnant women and their spouses who agreed to participate in the study and complete the follow-up survey were recruited. Considering that assisted reproductive technology (ART) may lead to overestimation of the prevalence of preterm birth and low birth weight, and there may be bias in estimating the impact of relevant risk factors. Therefore, the population using ART was not included in the analysis (*n* = 9,944). For the purpose of this study, we excluded pregnant women with twins or above (including the case that one of the original twins died naturally) (*n* = 661), with termination of pregnancy due to miscarriage, stillbirth and induced labor (*n* = 603), still pregnant at the end of follow-up (*n* = 2,870) or lost to follow-up (*n* = 831), as well as men with implausible measurements of height (<150 cm) (*n* = 56) or weight (<30 kg) (*n* = 89) were excluded. A total of 34,104 subjects were included in the final analysis (Figure 1).

In the process of recruiting study population, we used self-designed structured questionnaire to conduct the face-to-face survey. Before that, our investigators had been strictly trained to ensure the accuracy of the data. In our study, the sociodemographic characteristics and pre-pregnancy exposure history of women and their spouses were collected at 14–16 weeks of pregnancy. This part was completed in the corresponding pregnancy examination. The information of the pregnancy outcome of the offspring was collected 43–60 days postpartum, which was completed by querying the information of the electronic medical record system.

### Exposure and outcome

The paternal pre-pregnancy BMI classified according to the appropriate BMI of Asian population recommended by the WHO: <18.5 kg/m^2^ underweight; 18.5 to <23 kg/m^2^ normal weight; 23 to <27.5 kg/m^2^ overweight; and ≥27.5 kg/m^2^ obese (24). The paternal height and weight were measured when he wore light clothes and removed his shoes.

In this study, the outcomes of interest were preterm birth and low birth weight. Preterm birth was defined as delivery before 37 weeks of gestation, and low birth weight was defined as birth weight <2,500 g. Gestation weeks were estimated based on data from the most recent menstrual cycle, or by ultrasound if menstruation was irregular. To better understand the association between outcome and exposure, we further divided preterm birth into four groups according to gestational age: <28 gestational weeks for extremely preterm birth, 28–31 gestational weeks for very preterm birth, 32–33 gestational weeks for early preterm birth, and 34–36 gestational weeks for late preterm birth. In addition, we divided low birth weight into two groups based on fetal birth weight: <1,500 g for very low birth weight, and 1,500 to <2,500 g for low birth weight.

### Statistical analysis

This study was reported following the STROBE statement (25). In the analysis of the basic characteristics of the study population, measurement data were described by means of mean and standard deviation (*$\bar{x}$*±*s*), and *t*-test was used for comparison; categorical variables were described by percentage and compared by χ^2^ test or Fisher’s exact probability method. The associations between paternal pre-pregnancy BMI and preterm birth and low birth weight were analyzed using a binary Logistic regression model. Then we further divided preterm birth into four groups according to gestational age and low birth weight into two groups according to birth weight. Multinomial logistic regression model was used to further analyze the association between paternal pre-pregnancy BMI and preterm birth and low birth weight. Odds ratio (OR) and 95% confidence intervals (CIs) were used to describe and compare the associations between different levels of factors. In above analysis, Model 1 was crude OR. Model 2 was adjusted for paternal age, maternal age, maternal BMI, residence location, education level, nationality, history of smoking, drinking, betel nut consumption, drug use, and per capita monthly household income (when the outcome was preterm birth, the history of preterm birth was also adjusted).

To assess the stability of the results, we analyzed the interactions effect between paternal age, residence location, and ethnicity and paternal pre-pregnancy BMI. Subgroup analysis was further performed by paternal age, residence location, and ethnicity. Using full-term birth and normal birth weight as reference, a binary Logistic regression model was used to estimate the risk associated with paternal BMI and outcomes (preterm birth and low birth weight) for each subgroup after adjusting for other covariables except subgroup variables. In this study, EpiData version 3.1 (EpiData Association, Odense, Denmark) was used to establish the database by double entry to ensure the accuracy of the data. The data were statistically analyzed from 8 January 2022 to 20 January 2022 and the results were obtained. SPSS 26.0 software was used to analyze the data, and R 4.0.3 was used to draw the forest map. Two-sided *p* < 0.05 was considered statistically significant.

## Results

### Characteristics of participants

In this study, 13,724 (40.2%) people were classified as normal weight, 1,442 (4.2%) were underweight, 13,930 (40.9%) were overweight, and 5,008 (14.7%) were obese according to paternal pre-pregnancy BMI. The mean age of the study population was 31.0 ± 4.8 years. Characteristics of study population classified by BMI were shown in Table 1.

**TABLE 1:** Characteristics of the study population by BMI categories.

Characteristics	Total	Under weight	Normal weight	Over weight	Obese	*P*-value
Age, years	31.0 ± 4.8	28.3 ± 3.7	30.4 ± 4.8	31.5 ± 4.9	31.6 ± 4.7	< 0.001
Maternal age, years	31.1 ± 4.5	31.1 ± 4.4	31.1 ± 4.4	31.1 ± 4.6	31.1 ± 4.5	0.453
Maternal BMI, *n* (%)						0.429
Underweight	4,920 (14.4)	193 (13.4)	1,948 (14.2)	2,083 (15.0)	696 (13.9)	
Normal weight	21,056 (61.7)	906 (62.8)	8,474 (61.7)	8,559 (61.4)	3,117 (62.2)	
Overweight	6,963 (20.4)	296 (20.5)	2,831 (20.6)	2,805 (20.1)	1,031 (20.6)	
Obese	1,165 (3.4)	47 (3.3)	471 (3.4)	483 (3.5)	164 (3.3)	
Residence location, *n* (%)						< 0.001
Urban area	21,074 (61.8)	1,010 (70.0)	8,432 (61.4)	8,436 (60.6)	3,196 (63.8)	
Rural area	13,030 (38.2)	432 (30.0)	5,292 (38.6)	5,494 (39.4)	1,812 (36.2)	
Education level, *n* (%)						< 0.001
Junior high and below	4,138 (12.1)	120 (8.3)	1,834 (13.4)	1,368 (9.8)	816 (16.3)	
High school or technical secondary school	8,714 (25.6)	870 (60.3)	4,428 (32.3)	2,752 (19.8)	664 (13.3)	
College or bachelor degree	18,362 (53.8)	388 (26.9)	6,522 (47.5)	8,416 (60.4)	3,036 (60.6)	
Master degree or above	2,890 (8.5)	64 (4.4)	940 (6.8)	1,394 (10.0)	492 (9.8)	
Nationality, *n* (%)						< 0.001
Han nationality	33,000 (96.8)	1,412 (97.9)	13,260 (96.6)	13,566 (97.4)	4,762 (95.1)	
Minority nationality	1,104 (3.2)	30 (2.1)	464 (3.4)	364 (2.6)	246 (4.9)	
History of smoking, *n* (%)						< 0.001
NO	19,546 (57.3)	480 (33.3)	7,944 (57.9)	8,530 (61.2)	2,592 (51.8)	
YES	14,558 (42.7)	962 (66.7)	5,780 (42.1)	5,400 (38.8)	2,416 (48.2)	
History of drinking, *n* (%)						< 0.001
NO	25,550 (74.9)	1,100 (76.3)	10,540 (76.8)	10,346 (74.3)	3,564 (71.2)	
YES	8,554 (25.1)	342 (23.7)	3,184 (23.2)	3,584 (25.7)	1,444 (28.8)	
History of betel nut consumption, *n* (%)						< 0.001
NO	21,448 (62.9)	820 (56.9)	9,122 (66.5)	8,884 (63.8)	2,622 (52.4)	
YES	12,656 (37.1)	622 (43.1)	4,602 (33.5)	5,046 (36.2)	2,386 (47.6)	
History of drug use, *n* (%)						0.323
NO	33,938 (99.5)	1,432 (99.3)	13,650 (99.5)	13,872 (99.6)	4,984 (99.5)	
YES	166 (0.5)	10 (0.7)	74 (0.5)	58 (0.4)	24 (0.5)	
History of preterm birth, *n* (%)						0.438
NO	33,802 (99.1)	1,432 (99.3)	13,596 (99.1)	13,802 (99.1)	4,972 (99.3)	
YES	302 (0.9)	10 (0.7)	128 (0.9)	128 (0.9)	36 (0.7)	
Per capita monthly household income, RMB, *n* (%)						< 0.001
≤2,500	5,892 (17.3)	816 (56.6)	2,890 (21.1)	1,806 (13.0)	380 (7.6)	
2,500–5,000	18,206 (53.4)	420 (29.1)	7,082 (51.6)	7,930 (56.9)	2,774 (55.4)	
>5,000	10,006 (29.3)	206 (14.3)	3,752 (27.3)	4,194 (30.1)	1,854 (37.0)	

**FIGURE 2:**
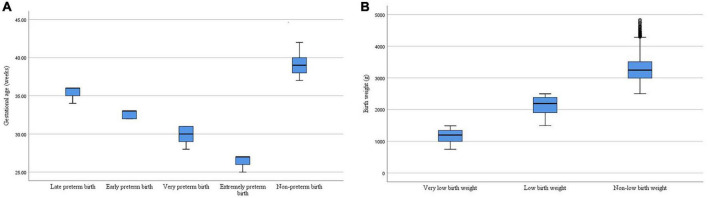
**(A)** The distribution of gestational age for different types of preterm birth. **(B)** The distribution of birth weight for different types of low birth weight.

In the total study population, the total preterm birth rate and total low birth weight rate were 11.85% (4,041/34,104) and 8.86% (3,020/34,104), respectively. We categorized preterm birth into four groups (extreme preterm birth; very preterm birth; early preterm birth; and late preterm birth) and low birth weight into two groups (very low birth weight; and low birth weight). The incidence of preterm birth and low birth weight and their subtypes among the offspring in this study were shown in Table 2. Among them, the median was 39 weeks for non-preterm birth, 27 weeks for extremely preterm birth, 30 weeks for very preterm birth, 33 weeks for early preterm birth, and 36 weeks for late preterm birth. The median was 3,245 g for non-low birth weight, 1,200 g for very low birth weight, and 2,190 g for low birth weight. The distribution of gestational age and birth weight for different types of preterm birth and low birth weight in this study were shown in Figure 2.

**TABLE 2:** Incidence of preterm birth and low birth weight and their subtypes in offspring (*n* = 34,104).

Outcomes	Number of cases	Incidence (95% CI)
The total preterm birth	4,041	11.85% (11.51–12.19%)
Extremely preterm birth	91	0.27% (0.21–0.32%)
Very preterm birth	581	1.70% (1.57–1.84%)
Early preterm birth	489	1.43% (1.31–1.56%)
Late preterm birth	2,880	8.44% (8.15–8.74%)
The total low birth weight	3,020	8.86% (8.55–9.16%)
Very low birth weight	645	1.89% (1.75–2.04%)
Low birth weight	2,375	6.96% (6.69–7.23%)

### Associations of paternal pre-pregnancy body mass index with preterm birth

In the overall study population, we found that the paternal pre-pregnancy overweight (aOR = 1.34, 95% CI: 1.25–1.45) or obesity (aOR = 1.26, 95% CI: 1.14–1.40) was associated with an increased risk of preterm birth after adjusting for potential confounding factors. When preterm birth was divided into four subgroups according to gestational age, each group showed that paternal overweight was associated with a significantly increased risk of preterm birth in their offspring. Among them, the risk of extremely preterm birth was the highest (aOR = 2.96, 95% CI: 1.77–4.94). Apparently, it was also found that paternal pre-pregnancy obesity was associated with an increased risk of preterm birth in each group (except for very extremely preterm birth, which may be due to small sample size) (Table 3).

**TABLE 3:** Odds ratio (95% CI) for the associations between paternal pre-pregnancy BMI and preterm birth and its subtypes.

Preterm birth	Case, *n* (%)	Under weight	Normal weight	Over weight	Obese
Overall	4,041 (100)				
Model 1		0.96 (0.80–1.15)	Ref.	1.28 (1.19–1.38)**	1.23 (1.12–1.36)**
Model 2		0.95 (0.79–1.15)	Ref.	1.34 (1.25–1.45)**	1.26 (1.14–1.40)**
Extremely preterm birth	91 (2.25)				
Model 1		1.35 (0.40–4.55)	Ref.	2.70 (1.64–4.47)**	1.47 (0.71–3.05)
Model 2		1.49 (0.43–5.12)	Ref.	2.96 (1.77–4.94)**	1.69 (0.80–3.55)
Very preterm birth	581 (14.38)				
Model 1		0.73 (0.42–1.26)	Ref.	1.66 (1.38–2.00)**	1.34 (1.04–1.74)*
Model 2		0.82 (0.47–1.42)	Ref.	1.79 (1.48–2.17)**	1.40 (1.08–1.83)*
Early preterm birth	489 (12.10)				
Model 1		1.05 (0.64–1.71)	Ref.	1.43 (1.16–1.74)*	1.36 (1.04–1.78)*
Model 2		1.05 (0.64–1.74)	Ref.	1.55 (1.26–1.91)**	1.48 (1.12–1.96)*
Late preterm birth	2,880 (71.27)				
Model 1		0.98 (0.80–1.20)	Ref.	1.16 (1.07–1.27)*	1.19 (1.06–1.33)*
Model 2		0.99 (0.80–1.22)	Ref.	1.20 (1.10–1.31)**	1.20 (1.07–1.35)*

Model 1 was crude OR. Model 2 was adjusted for paternal age, maternal age, maternal BMI, residence location, education level, nationality, history of smoking, history of drinking, history of betel nut consumption, history of drug use, history of preterm birth, and per capita monthly household income.

**p < 0.001; *p < 0.05.

### Associations of paternal pre-pregnancy body mass index with low birth weight

When analyzing the association between paternal pre-pregnancy BMI and low birth weight in the offspring, it was found that paternal overweight (aOR = 1.60, 95% CI: 1.46–1.74) or obesity (aOR = 1.40, 95% CI: 1.25–1.58) was associated with an increased incidence of low birth weight in offspring in the overall study population after adjusting for potential confounding factors. In contrast, paternal pre-pregnancy underweight may be a protective factor for low birth weight in offspring (aOR = 0.73, 95% CI: 0.57–0.92). A similar association pattern was observed when low birth weight was divided into two subgroups based on birth weight. Paternal overweight or obesity was also associated with low birth weight in offspring in both subgroups. However, it was not found that paternal pre-pregnancy underweight was associated with very low birth weight infants (Table 4).

**TABLE 4:** Odds ratio (95% CI) for the associations between paternal pre-pregnancy BMI and low birth weight and its subtypes.

Birth weight	Case, *n* (%)	Under weight	Normal weight	Over weight	Obese
Overall	3,020 (100)				
Model 1		0.74 (0.58–0.93)*	Ref.	1.45 (1.34–1.58)**	1.34 (1.20–1.51)**
Model 2		0.73 (0.57–0.92)*	Ref.	1.60 (1.46–1.74)**	1.40 (1.25–1.58)**
Very low birth weight	645 (21.36)				
Model 1		0.74 (0.46–1.19)	Ref.	1.31 (1.10–1.56)*	1.31 (1.04–1.65)*
Model 2		0.78 (0.48–1.28)	Ref.	1.40 (1.17–1.68)**	1.38 (1.09–1.75)*
Low birth weight	2,375 (78.64)				
Model 1		0.74 (0.57–0.96)*	Ref.	1.50 (1.36–1.64)**	1.36 (1.19–1.54)**
Model 2		0.74 (0.56–0.97)*	Ref.	1.64 (1.49–1.81)**	1.50 (1.31–1.71)**

Model 1 was crude OR. Model 2 was adjusted for paternal age, maternal age, maternal BMI, residence location, education level, nationality, history of smoking, history of drinking, history of betel nut consumption, history of drug use, and per capita monthly household income.

**p < 0.001; *p < 0.05.

### Subgroup analysis

**FIGURE 3:**
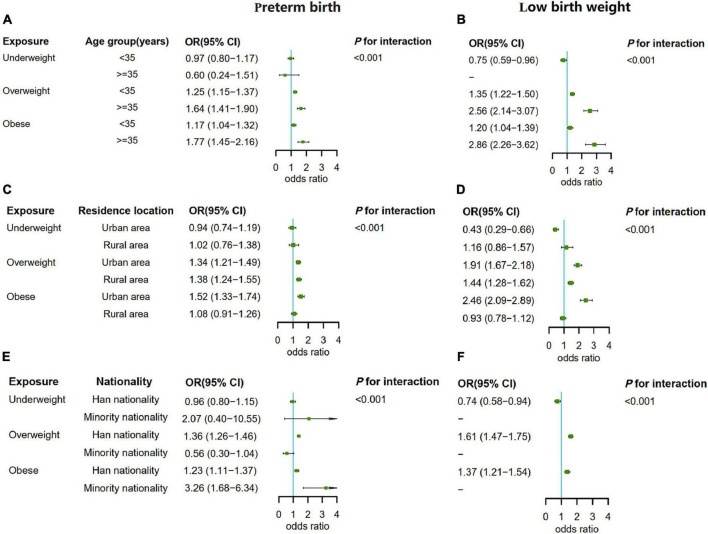
**(A)** The subgroup analysis by paternal age when the outcome was preterm birth. **(B)** The subgroup analysis by paternal age when the outcome was low birth weight. **(C)** The subgroup analysis by residence location when the outcome was preterm birth. **(D)** The subgroup analysis by residence location when the outcome was low birth weight. **(E)** The subgroup analysis by nationality when the outcome was preterm birth. **(F)** The subgroup analysis by nationality when the outcome was low birth weight.

Through statistical analysis, there was an interaction effect between paternal pre-pregnancy BMI and age, residence location and nationality (all *p* for interaction <0.001). Then we conducted a subgroup analysis of the association between paternal pre-pregnancy BMI and preterm birth and low birth weight. In the three subgroups, paternal pre-pregnancy overweight or obesity at all levels were associated with an increased risk of preterm birth and low birth weight in the offspring (except rural area and minority nationality) after adjusting for the confounding effects of other covariables other than the subgroup factors. For rural area and minority nationality participants, the risk association was not found to be statistically significant, which may be related to small exposure, and the small sample size of each group in the subgroup analysis (the sample size of low birth weight of offspring in the minority group with underweight and normal weight was 0). Overall, the relationship between paternal overweight or obesity and preterm birth and low birth weight remained in the same direction, suggesting a stable risk association. In addition, we found that the risk of preterm birth and low birth weight was higher in the offspring of fathers with overweight or obesity in age ≥35 years than in the offspring of fathers with overweight or obesity in age <35 years. Moreover, the risk of preterm birth of fathers with obesity (aOR = 3.26, 95% CI: 1.68–6.34) in minority was higher than that of fathers with obesity (aOR = 1.23, 95% CI: 1.11–1.37) in the Han nationality (Figure 3).

## Discussion

We conducted a prospective cohort study using information about pregnant women and their spouses in Hunan maternal and child health hospital. After adjusting for maternal and paternal confounders, we found that paternal pre-pregnancy overweight and obesity were significantly associated with an increased risk of preterm birth and low birth weight in offspring. When we divided preterm birth into four groups (extreme preterm birth; very preterm birth; early preterm birth; and late preterm birth) and low birth weight into two groups (very low birth weight; and low birth weight), these associations still existed. Subgroup analysis (paternal age, residence location, and nationality) also showed that the risk associations were stable. Therefore, our study suggested that paternal pre-pregnancy overweight and obesity may be risk factors for preterm birth and low birth weight in offspring.

Our study has pointed out a key problem. There may be a close association between paternal factors and pregnancy outcomes. At present, most studies only focus on the impact of maternal exposure factors on the health of offspring, which has played an important role in health education and publicity. However, the incidence of the diseases are still high, suggesting that we still need to pay attention to other causes, such as father related factors. Paternal obesity has been reported to increase the risk of preeclampsia, SGA, birth defects and preterm birth (19, 26–29). According to the study results of He et al., compared with the pre-pregnancy normal weight, the corresponding ORs for paternal overweight and obesity were: preterm birth (1.12; 1.10–1.14 vs. 1.24; 1.20–1.28), low birth weight (1.10; 1.05–1.15 vs. 1.29; 1.20–1.40), which was consistent with our results (29). In addition, the BMI classification and population (Chinese) in the two studies were the same. However, the results of another epidemiological survey in China were contrary to us. They believed that high paternal BMI (>24 kg/m^2^) was associated with a low risk of low birth weight, preterm birth and SGA (30). A possible reason for this difference is the use of different BMI cut-off points for fathers. However, the larger sample size gives us confidence. Moreover, this association persisted when we subdivided gestational age and birth weight. There were also studies suggesting that paternal pre-pregnancy BMI was not associated with preterm birth and low birth weight in their offspring (21, 22, 31). However, these data were usually based on retrospective studies, which are open to recall bias.

One interesting finding of our study is that we found a significant interaction between paternal pre-pregnancy BMI and age, residence location and nationality (*p* value for interaction was significant) in relation to the risk of preterm birth and low birth weight. We further performed subgroup analyses and found that the risk of preterm birth and low birth weight was higher in the offspring of fathers with overweight or obesity in age ≥35 years than the offspring of fathers with overweight or obesity in age <35 years. It may reveal that paternal age was also a risk factor for preterm birth and low birth weight in the offspring, which was similar to most of the studies (32–34). The mechanism may be attributed to age-related sperm abnormalities or chromosomal mutations (35). One important reason for sperm abnormality is the toxic effect of reactive oxygen species (ROS). With the increase of age, the antioxidant capacity of the human body gradually declines, and the ROS produced cannot be removed in time, resulting in a large amount of ROS accumulation. During certain key stages of spermatogenesis, sperm are highly vulnerable to ROS, resulting in reduced sperm count and quality in older men. In addition, the risk of preterm birth in the offspring of fathers with obesity in minority was higher than that of fathers with obesity in the Han nationality. This may be related to genetics, eating habits and lifestyle.

The biological mechanisms underlying the effects of paternal pre-pregnancy overweight and obesity on preterm birth and low birth weight infants are unclear. It may be affected by aggravating endocrine disorders and sperm quality (36). It is known that maintaining physiological balance of the hypothalamic-pituitary-gonadal (HPG) axis is a prerequisite for spermatogenesis (37). The pituitary gland releases follicle-stimulating hormone (FSH) and luteinizing hormone (LH) were reported to be negatively associated with BMI in obese men (38). The change of HPG axis leads to low gonadotropin, high estrogen and low androgen, which will affect male reproductive function in the long term (36). In addition, obesity can lead to changes in sperm DNA, reduced mitochondrial function and increased inflammation. Dupont et al. found that men with high BMI had a significantly higher risk of sperm DNA fragmentation index (39). Mitochondria provide sufficient energy for the spermatogenesis process and the combination with the egg after ejaculation. However, obesity destroys mitochondria in sperm by inducing oxidative stress, which leads to sperm morphological defects and reduced motility (40, 41). Another study also found that the inflammatory factors in the seminal plasma of obese men increased, which may affect the quality of semen (42). It is important to note that the pathologic activation of the inflammatory signal cascade is the result of an infection or sterile inflammatory trigger, which may induce spontaneous preterm birth when seminal fluid with more inflammatory characteristics arrives in the uterine environment (43).

The strength of our study was the large sample size. We also performed a more comprehensive subgroup analysis, but it still had limitations. First of all, the study population was all from the same hospital, and the sample sources may be concentrated in a certain group of population. This may affect the representativeness of the sample and thus affect extrapolation. Secondly, paternal height and weight were self-reported, which increases the risk of bias. However, we also measured the paternal height and weight again on the spot to reduce his recall bias. Thirdly, although the age and BMI of mothers were adjusted in this study, the dietary patterns of fathers and mothers were not collected, so their influence on the study results cannot be completely excluded. Fourthly, Matsushita et al. pointed out that maternal pre-pregnancy obesity was significantly associated with the risk of preterm birth, but the risk varies according to maternal age, race or ethnic group (14). A similar statement may also exist in the association between paternal pre-pregnancy BMI and offspring’s birth health (44). Fifthly, the fetuses in this study all had live births. Inevitably, there were still some women who choose to terminate a pregnancy due to miscarriage, stillbirth, and induction of labor, and this part of the missed events may result in competing risk in the results of this study. These limitations highlight the urgent need for larger prospective studies in different populations to determine the causes of preterm birth and low birth weight in order to better prevent disease. In general, our results still have strong scientific and theoretical significance.

## Conclusion

We have shown that paternal pre-pregnancy overweight and obesity were associated with an increased risk of preterm birth and low birth weight in offspring, and this appeared to be independent of maternal factors. The new evidence we provided may represent a critical step toward preventing and predicting preterm birth and low birth weight. However, more prospective studies with larger samples are needed to verify the authenticity of the results and explore the underlying mechanisms. We advocate the community to call on men to pay more attention to weight management before pregnancy in order to reduce the adverse effects of overweight and obesity on the health of their offspring.

## Data availability statement

The raw data supporting the conclusions of this article will be made available by the authors, without undue reservation.

## Ethics statement

This study was approved by the Ethics Committee of Xiangya School of Public Health, Central South University, and registered in the Chinese Clinical Trial Registry (registration number: ChiCTR1800016635). The patients/participants provided their written informed consent to participate in this study.

## Author contributions

MS conceptualized the study and drafted the initial manuscript. MS, SZ, and LC analyzed and explained the data. JD, SZ, JL, YHL, LC, YPL, JW, XS, and JS have collected the data. TW, PZ, and JQ conceptualized and designed the study and critically reviewed and revised the manuscript. All authors contributed to the article and approved the final version of the manuscript.
